# Artificial intelligence assessment for early detection and prediction of renal impairment using electrocardiography

**DOI:** 10.1007/s11255-022-03165-w

**Published:** 2022-04-11

**Authors:** Joon-myoung Kwon, Kyung-Hee Kim, Yong-Yeon Jo, Min-Seung Jung, Yong-Hyeon Cho, Jae-Hyun Shin, Yoon-Ji Lee, Jang-Hyeon Ban, Soo Youn Lee, Jinsik Park, Byung-Hee Oh

**Affiliations:** 1 Medical Research Team, Medical AI, co., Seoul, South Korea; 2 Artificial Intelligence and Big Data Research Center, Sejong Medical Research Institute, Bucheon, South Korea; 3 Medical R&D Center, Body Friend, co, Seoul, South Korea; 4 Department of Critical Care and Emergency Medicine, Incheon Sejong Hospital, 20, Gyeyangmunhwa-ro, Gyeyang-gu, Incheon, Republic of Korea; 5 Division of Cardiology, Department of Internal Medicine, Incheon Sejong Hospital, Cardiovascular Center20, Gyeyangmunhwa-ro, Gyeyang-gu, Incheon, Republic of Korea

**Keywords:** Renal insufficiency, Deep learning, Electrocardiography, Artificial intelligence

## Abstract

**Purpose:**

Although renal failure is a major healthcare burden globally and the cornerstone for preventing its irreversible progression is an early diagnosis, an adequate and noninvasive tool to screen renal impairment (RI) reliably and economically does not exist. We developed an interpretable deep learning model (DLM) using electrocardiography (ECG) and validated its performance.

**Methods:**

This retrospective cohort study included two hospitals. We included 115,361 patients who had at least one ECG taken with an estimated glomerular filtration rate measurement within 30 min of the index ECG. A DLM was developed using 96,549 ECGs of 55,222 patients. The internal validation included 22,949 ECGs of 22,949 patients. Furthermore, we conducted an external validation with 37,190 ECGs of 37,190 patients from another hospital. The endpoint was to detect a moderate to severe RI (estimated glomerular filtration rate < 45 ml/min/1.73m^2^).

**Results:**

The area under the receiver operating characteristic curve (AUC) of a DLM using a 12-lead ECG for detecting RI during the internal and external validation was 0.858 (95% confidence interval 0.851–0.866) and 0.906 (0.900–0.912), respectively. In the initial evaluation of 25,536 individuals without RI patients whose DLM was defined as having a higher risk had a significantly higher chance of developing RI than those in the low-risk group (17.2% vs. 2.4%, *p* < 0.001). The sensitivity map indicated that the DLM focused on the QRS complex and T-wave for detecting RI.

**Conclusion:**

The DLM demonstrated high performance for RI detection and prediction using 12-, 6-, single-lead ECGs.

**Supplementary Information:**

The online version contains supplementary material available at 10.1007/s11255-022-03165-w.

## Introduction

Renal impairment (RI), including chronic kidney disease and acute kidney injury, is an important contributor to morbidity and mortality. Globally, in 2017, over 1.2 million people died from RI [1]. The treatment cost for RI increases with the availability of renal replacement techniques, resulting in a life-saving but expensive treatment in the long term for patients with end-stage kidney disease [1]. The number of people currently receiving renal replacement therapy exceeds 2.5 million and is projected to double to approximately 5.4 million by 2030 [2]. RI is emerging as a major healthcare burden worldwide, and 2.3–7.1 million adults die from a lack of access to renal replacement therapy [2]. The cornerstone to prevent irreversible progression of RI and initiate appropriate treatment is the early detection of RI [3, 4].

However, most cases of mild kidney function decline are asymptomatic, and the symptoms for the progression of the disease are vague and nonspecific [5]. A diagnostic test for renal failure includes a laboratory examination to measure the creatinine and blood urea nitrogen and calculate the glomerular filtration rate [6]. Laboratory tests are invasive, expensive, and require specialized equipment and infrastructure, such as trained medical staff for blood sampling and a hematologic analysis machine for assessment with biochemical reagents. Therefore, detecting RI in daily living is impossible, and screening for RI is difficult in low-income countries [7].

RI is associated with electrolyte imbalance, volume overload, and hypertension and also affects cardiac function [8–10]. RI is a known cause of diastolic dysfunction, left ventricular hypertrophy, arrhythmia, and heart failure and is associated with increased cardiovascular mortality [11, 12]. In several studies, RI was shown to change the morphology of an electrocardiogram (ECG), and researchers suggested that the alteration of cardiac function and electrolyte imbalance affects an ECG [13–15]. However, it is not easy to detect such subtle and non-linear ECG changes; hence, the current state of the ECG is not useful for detecting RI. Screening and detecting RI with an ECG would be useful because patients suspected to have RI could be referred for confirmatory laboratory tests.

In this study, we aimed to develop and validate a deep learning-based artificial intelligence model (DLM) for detecting RI using ECG. Deep learning has previously been used in the medical field to identify lesions and is currently used to analyze ECGs to diagnose heart failure, valvular heart disease, anemia, and coronary artery disease [16–24]. We hypothesized that a DLM could effectively screen for RI.

## Methods

### Study design and population

We conducted a retrospective, multicenter, diagnostic study in which a DLM was developed using ECGs, and then, it was internally and externally validated. We excluded individuals with missing demographic, ECG, and laboratory examination information. Data from Sejong General Hospital (SGH) were used for development and internal validation. In SGH, we identified patients with at least one standard digital 10-s 12-lead ECG acquired in the supine position within the study period (October 1, 2016 to August 31, 2020) and at least one renal laboratory panel for serum creatinine and blood urea nitrogen obtained within 30 min of the index ECG. The individuals who visited SGH for inpatient, outpatient, emergency, and health checkup clinic were the study population for the development and internal validation datasets of the DLM. As shown in Supplementary Figure S1, patients who underwent a follow-up laboratory examination after an initial evaluation were assigned to an internal validation dataset. Patients who had no follow-up laboratory exam were assigned to a development dataset that was used to develop the DLM. Subsequently, we evaluated the accuracy of the DLM using the internal validation dataset. Data from Mediplex Sejong Hospital (MSH) were used for external validation. We identified the patients who were admitted to MSH during the study period (March 1, 2017 to August 31, 2020) and who had at least one ECG and at least one renal laboratory panel for serum creatinine and blood urea nitrogen obtained within 30 min of the index ECG. Because the purpose of the validation data was to assess the accuracy of the algorithm, we used only one ECG of each patient for the internal and external validation datasets, i.e., the ECG obtained closest to the patient’s laboratory exam during the study period.

This study was approved by the institutional review boards of the SGH and MSH. Clinical data, including digitally stored ECGs, the laboratory examination results of the renal panel, age, and sex of patients were obtained from both hospitals. Both institutional review boards waived the need for informed consent because of the retrospective nature of the study using fully anonymized ECG and health data and causing minimal harm.

### Procedures

The predictor variables used were ECG, age, and sex. Digitally stored 12-lead ECG data, amounting to 5000 data points for each lead, were recorded for 10 s (500 Hz). We removed 1 s each at the beginning and end of each ECG because they had more artifacts than other parts. Therefore, the length of each ECG was 8 s (4000 data points). We created a dataset using the entire 12-lead ECG data. In addition, we used partial datasets from 12-lead ECG data, such as limb six-lead and single lead (I). We selected the sets of leads because these leads could easily be recorded by wearable and pad devices in contact with the hands and legs. Consequently, when we developed and validated an DLM using 12-lead ECGs, we used a dataset of two-dimensional (2D) data of 12 × 4000 data points. When we developed and validated an algorithm using six-lead ECGs, we used datasets of 6 × 4000 data points, and when using single-lead ECGs, we used datasets of 1 × 4000 data points.

The primary endpoint of this study was a moderate to severe RI, which was defined as the estimated glomerular filtration rate (eGFR) under 45 ml/min per 1.73 m^2^. The eGFR was calculated using the modification of diet in the renal disease study equation (eGFR = 175 × (serum creatinine)^−1.154^ × (age)^−0.203^ × 0.742 [if female] × 1.212 [if Black]) [25, 26]. The secondary endpoint was a mild to severe RI, which was defined as an eGFR under 60 ml/min per 1.73 m^2^.

**Fig. 1:**
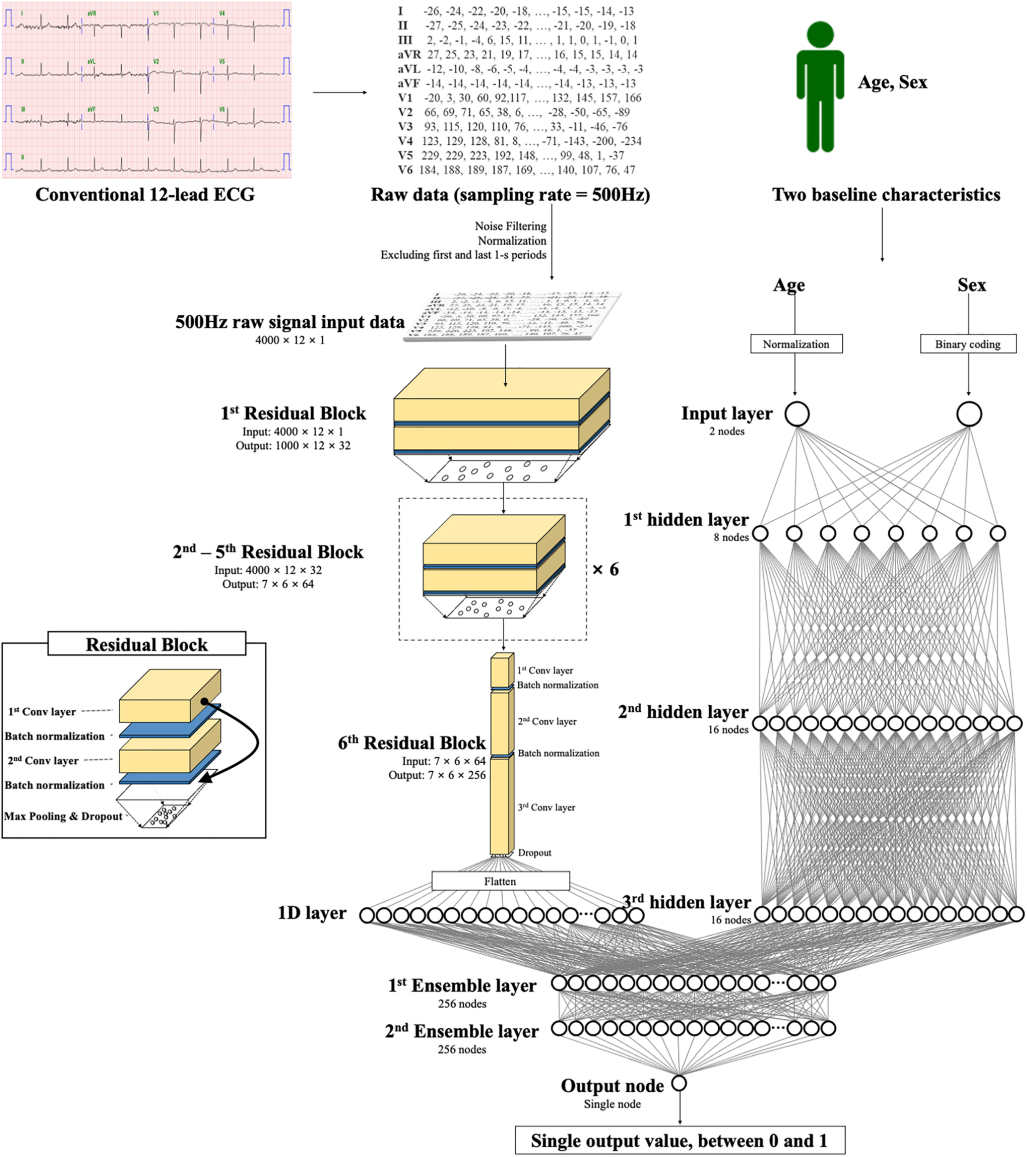
Architecture of deep learning based model for detecting renal impairment. Legend: 1D denotes 1-dimension and Conv convolution neural network

The DLM was developed using several hidden layers of neurons to learn complex hierarchical non-linear representations from the data. A residual block with six stages included two convolution layers, two batch normalizations, one max-pooling, and one dropout layer repeated, as shown in Fig. 1. We used 1 × 4 max-pooling layers between blocks 1 and 4 and 2 × 4 max-pooling layers between blocks 4 and 6. The last convolutional layer of the residual block was connected to a flattened layer, which was fully connected to the one-dimensional (1D) layer composed of 256 nodes. The input layer of epidemiology data (age and sex) was concatenated with the 1D layer. Two fully connected 1D layers were connected to the output node, which was composed of one node. The output node used a softmax function as an activation function because the output of the softmax function was between 0 and 1. The architecture of the DLM was evaluated and verified using a grid search. We developed additional DLM using limb six-lead and single-lead (I) ECGs.

### Statistical analysis

Continuous variables were presented as mean values (standard deviation, SD) and compared using the unpaired Student’s *t*-test or Mann–Whitney *U* test (if variables were found to be not normally distributed). We checked the homogeneity of the variance when using the unpaired Student’s *t*-test. Categorical variables were expressed as frequencies and percentages and compared using the χ2 test. At each input (ECG) of validation data, the DLM calculated the possibility of a primary endpoint in the range from 0 (a non-moderate to severe RI) to 1 (a moderate to severe RI). To verify the DLM performance, we compared the possibility calculated by the DLM with the presence of a moderate to severe RI in the internal and external validation datasets. To achieve this, we used the area under the receiver operating characteristic curve (AUC). The performance of the DLM for detecting the secondary endpoint, i.e., a mild to severe RI, was similarly verified using AUC. We applied the cutoff point to internal and external validation data to calculate sensitivity, specificity, positive predictive value (PPV), and negative predictive value (NPV). Sensitivity, specificity, PPV, and NPV were confirmed at the operating point from Youden J statistics in the development data [27]. Exact 95% confidence intervals (CIs) were used for all measures of diagnostic performances, except for AUC. The CIs for AUC were determined based on the Sun and Su optimization of the De-long method using the pROC package in R (The R Foundation for Statistical Computing, Vienna, Austria). We evaluated the *p*-value of the difference between the AUCs using the bootstrap methods. The bootstrap operation for *p*-value was performed with non-parametric resampling and the percentile method. The number of bootstrap replicates was 2000, which was recommended by Carpenter and Bithell [28]. A significant difference in patient characteristics was defined as a two-sided *p* value of less than 0.001. We also calculated effect size of results. The effect size was calculated using the bootstrap method. We defined effect sizes of 0.2, 0.5, 0.8 as indicative of small, moderate, and large clinical changes [29]. Statistical analyses were computed using R software, version 3.4 In addition, we used PyTorch’s open-source software library as the backend and Python (version 3.6) for the analysis.

### Visualizing developed XDM for interpretation

To understand the developed model and compare it to existing medical knowledge, it was necessary to identify a region that had a significant effect on the decision of the developed DLM. We employed a sensitivity map using a saliency method [30, 31]. The map was computed using the first-order gradients of the classifier probabilities with respect to the input signals; if the probability of a classifier was sensitive to a specific region of the signal, the region would be considered as significant in the model. In other words, we verified the region of ECG that was associated with RI using a sensitivity map. We used a gradient class activation map as a sensitivity map, and we guided the gradient backpropagation method. Further, we verified the variable importance of ECG features, age, and sex in logistic regression, random forest, and deep learning using the deviance difference, mean decreased Gini, and relative importance based on Garson’s algorithm, respectively [32]. A logistic regression model was derived using the maximum likelihood method to calculate coefficients via “glmulti” packages in R (R Development Core Team, Vienna, Austria). We used iteratively reweighted least squares (IWLS) to fit the final model. In logistic regression, R-squared was calculated using the Cox and Snell method. The random forest model consisted of 20,000 decision trees using the “randomForest” package in R. Additionally, the AUROC between the prognostic score and classification of RI in logistic regression and random forest in the test dataset were also confirmed. In logistic regression model, residual standard error and adjusted R-squared were 0.2366 and 0.0654.

### Verifying DLM performance to predict RI development as subgroup analysis

We hypothesized that the ECGs would display subtle abnormal patterns in the pre-RI phase and that the developed DLM would classify certain cases as abnormal, yielding a false positive test (a study subject classified as having RI but considered as non-RF) as the initial result. We conducted a subgroup analysis of patients who underwent follow-up laboratory examinations in the internal and external validation datasets. The difference in date between the initial and follow-up echocardiography data was over 14 days. Among those patients, we verified the development of RI in patients who were initially considered non-RI, whose eGFR was 60 ml/min per 1.73 m^2^ or over. The DLM was categorized into high- and low-risk groups based on the risk score using cutoff values, which were determined using the Youden’s J statistic with the development dataset [27]. We used the Kaplan–Meier method to analyze the RI development over 24 months.

## Results

The eligible population included 78,188 and 37,201 patients from SGH and MSH, respectively. We excluded 17 and 11 patients (from SGH and MSH, respectively) because of missing clinical information (age and sex), laboratory evaluation information, or ECG data (Supplementary Figure S1). The study included a total of 115,361 patients, of which 7362 patients had a moderate to severe RI. The DLM was developed using a development dataset of 96,549 ECGs of 55,222 patients from SGH (47.9%). Then, the performance of the algorithm was verified using 22,949 ECGs of 22,949 patients from SGH (19.9%) in the internal validation dataset and 37,190 ECGs of 37,190 patients from MSH (32.2%) in the external validation dataset. In moderate to severe RI, the ECGs had prolonged QRS duration, prolonged QTc, rightward T-wave axis, prolonged PR interval, and tachycardia (Table 1).

**Table 1:** Baseline characteristics

Characteristic	Development and internal validation dataset (Hospital A) *n* = 78,171					External validation dataset (Hospital B) *n* = 37,190				*P*^c^
	Non-renal impairment	Renal impairment	Effect size (95% CI)^a^	*p*^b^		Non-renal impairment	Renal impairment	Effect size (95% CI)^a^	*p*^b^	
Study population, *n* (%)	72,393 (92.6)	5778 (7.4)				35,606 (95.7)	1584 (4.3)			<0.001
Age, years, mean (SD)	80.18 (15.04)	64.28 (10.49)	−1.08 (−1.11 to −1.05)	<0.001		86.32 (16.20)	65.84 (12.55)	−1.21 (−1.26 to −1.16)	<0.001	<0.001
Male, *n*, (%)	39,026 (53.9)	2,384 (41.3)		<0.001		17,386 (48.8)	703 (44.4)		0.001	<0.001
Heart rate, bpm (SD)	71.26 (16.57)	78.76 (22.37)	0.44 (0.41–0.47)	<0.001		71.25 (15.66)	81.51 (22.85)	0.64 (0.59–0.69)	<0.001	0.262
PR interval, ms, mean (SD)	171.36 (29.03)	181.26 (41.11)	0.33 (0.30–0.36)	<0.001		166.07 (25.91)	176.68 (36.38)	0.40 (0.35–0.45)	<0.001	<0.001
QT interval, ms, mean (SD)	406.32 (40.18)	411.77 (55.95)	0.13 (0.10–0.16)	<0.001		401.06 (37.32)	403.23 (56.70)	0.06 (0.01–0.11)	0.027	<0.001
QRS duration, ms, mean (SD)	96.45 (17.46)	102.44 (24.40)	0.33 (0.30–0.36)	<0.001		94.42 (14.45)	100.18 (23.00)	0.39 (0.34–0.44)	<0.001	<0.001
QTc, ms, mean (SD)	436.54 (33.07)	460.20 (42.46)	0.70 (0.67–0.73)	<0.001		431.51 (30.68)	458.43 (42.60)	0.86 (0.81–0.91)	<0.001	<0.001
P axis, mean (SD)	43.58 (29.51)	41.71 (40.63)	−0.06 (−0.09 to −0.03)	<0.001		44.35 (27.26)	41.35 (35.47)	−0.11 (−0.16 to −0.06)	<0.001	<0.001
R axis, mean (SD)	38.59 (43.52)	31.86 (52.46)	−0.15 (−0.18 to −0.13)	<0.001		41.35 (38.96)	26.28 (47.25)	−0.38 (−0.43 to −0.33)	<0.001	<0.001
T axis, mean (SD)	43.92 (46.89)	68.05 (69.89)	0.49 (0 47–0.52)	<0.001		38.31 (35.45)	68.18 (64.66)	0.80 (0.75–0.85)	<0.001	<0.001
EGFR, mean (SD)	88.08 (20.48)	29.79 (11.83)	−2.92 (−2.95 to −2.89)	<0.001		97.73 (22.24)	26.29 (13.37)	−3.26 (−3.31 to −3.20)	<0.001	<0.001

^a^Standardized mean difference or Odds ratio

^b^The alternative hypothesis for this *p* value was that there was a difference between the renal impairment and non-renal impairment

^c^The alternative hypothesis for this *p* value was that there is a difference between hospital A (derivation and internal validation data group) and hospital B (external validation group) for each variable

**Fig. 2:**
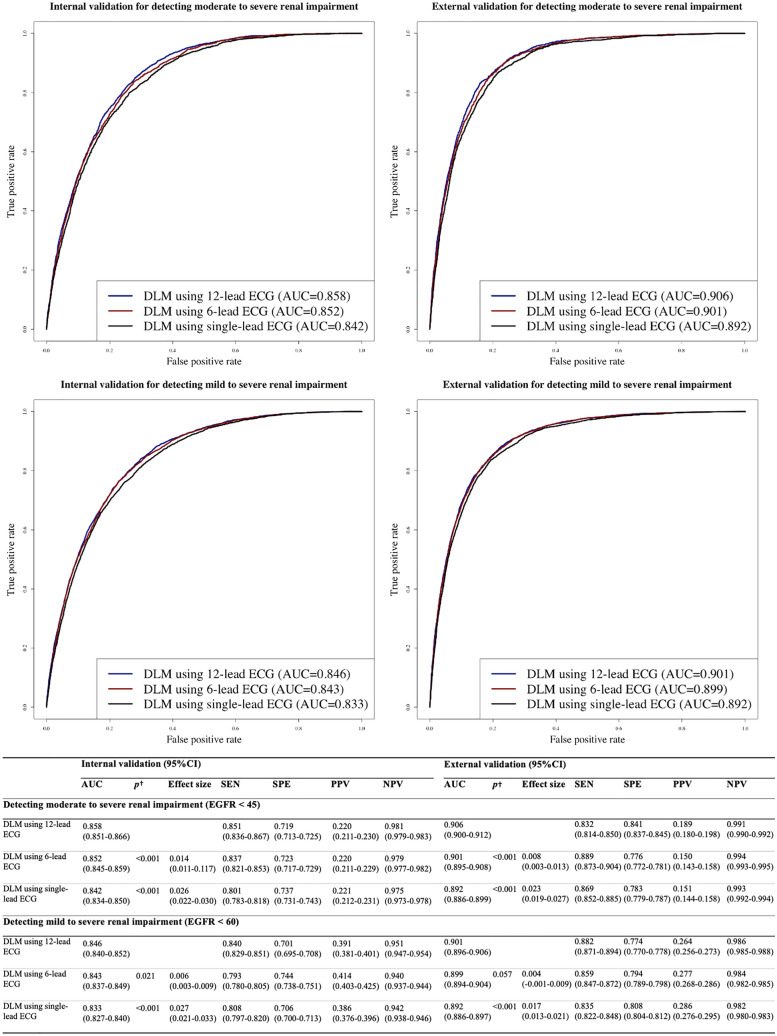
Performances of deep learning-based model for detecting renal impairment. Legend: †The alternative hypothesis for this *p* value was that there was a difference of AUC between the 12-lead ECG model and others. AUC denotes area under the receiver operating characteristic curve, ECG electrocardiography, EGFR estimated glomerular filtration rate, NPV negative predictive value, PPV positive predictive value, SEN sensitivity, and SPE specificity

During internal and external validations, the AUC of the DLM for detecting a moderate to severe RI as the primary endpoint using 12-lead ECGs was 0.858 (95% CI 0.851–0.866) and 0.906 (95% CI 0.900–0.912), respectively (Fig. 2). The AUC of the DLM for detecting a moderate to severe RI using six-lead ECGs during internal and external validations was 0.852 (95% CI 0.845–0.859) and 0.901 (95% CI 0.895–0.908), respectively. The AUC of the DLM using single-lead ECGs during internal and external validations were 0.842 (95% CI 0.834–0.850) and 0.892 (95% CI 0.886–0.899), respectively. During internal and external validation, the p-values for differences of AUC of the DLM for detecting a moderated to severe RI using 12 leads ECG and other leads ECG were <0.001. During internal and external validation, the effect size between AUCs of the DLM for detecting a moderated to severe RI between the 12-lead ECG model and 6-lead ECG model was 0.014 and 0.008, respectively. During internal and external validation, the effect size between AUCs of the DLM for detecting a moderated to severe RI between the 12-lead ECG model and 6-lead ECG model was 0.026 and 0.023, respectively. During internal and external validations, the AUC of the DLM for detecting a mild to severe RI as the secondary endpoint using 12-lead ECGs was 0.846 (95% CI 0.840–0.852) and 0.901 (95% CI 0.896–0.906), respectively (Fig. 2).

**Fig. 3:**
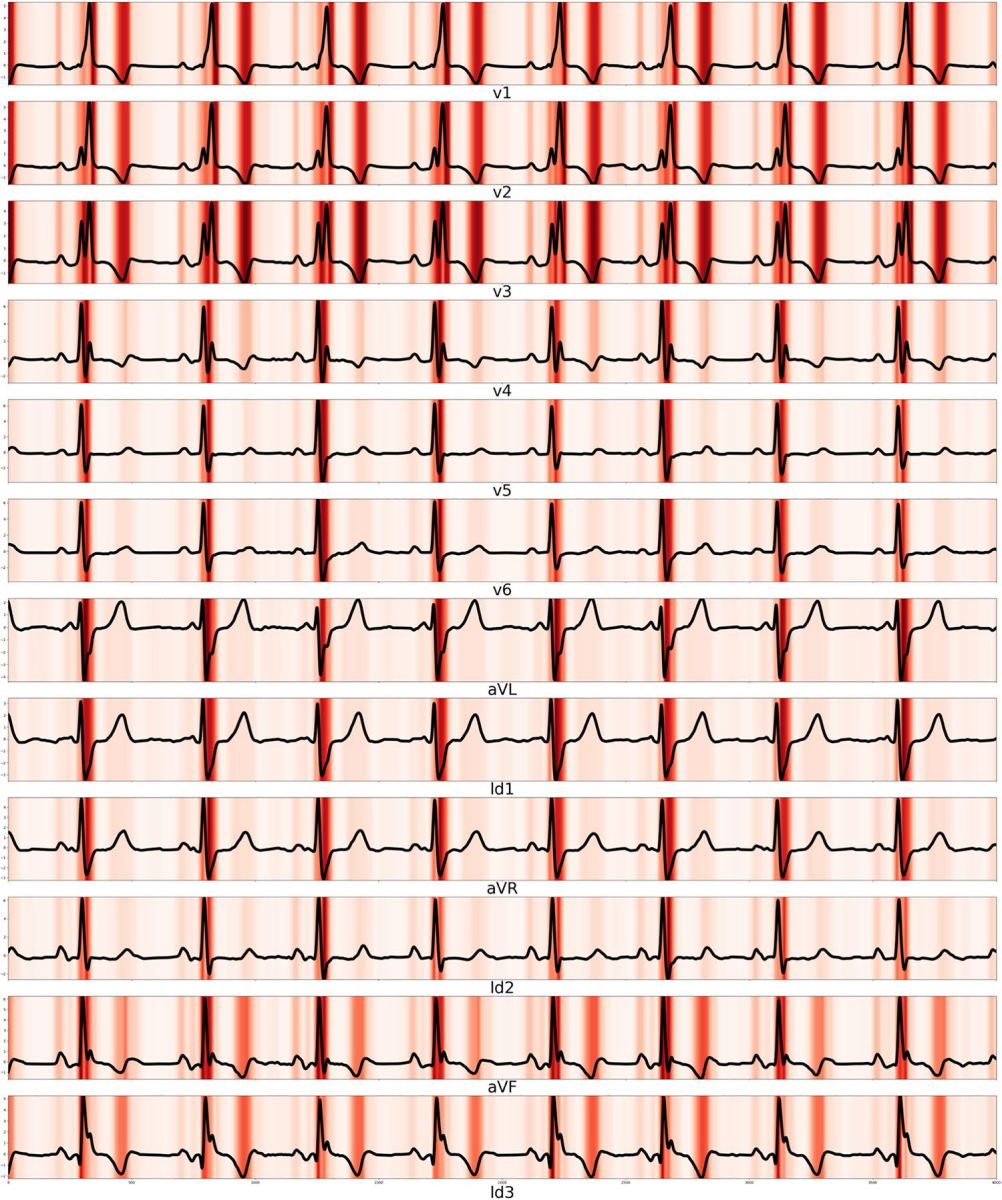
Sensitivity map of deep learning based model for detecting renal impairment

The DLM described the important ECG region for RI detection. As shown in Fig. 3, the DLM focused on the QRS complex and T wave for detecting RI. As shown in Table 2, the variable importance differed for each prognostic model. The logistic regression and random forest used the T-wave axis and the DLM used the QT interval as an important predictive variable. In the logistic regression model, the residual standard error and adjusted R-squared were 0.2366 and 0.0654, respectively.

**Table 2:** Variable importance for detecting renal impairment

Rank	Logistic regression (defiance difference)	Random forest (mean decrease Gini)	Deep learning (relative importance)
1	Age (−4789)	Age (1587.1)	Age (0.173)
2	Heart rate (−854)	T wave axis (1411.1)	QT interval (0.141)
3	T-wave axis (−366)	R wave axis (1216.6)	Heart rate (0.134)
4	QT interval (−286)	P wave axis (1211.1)	T wave axis (0.104)
5	PR interval (−109)	QT interval (1192.7)	P wave axis (0.097)
6	P wave axis (−7)	PR interval (1136.5)	QRS duration (0.094)
7	QRS duration (−5)	QRS duration (1093.1)	PR interval (0.093)
8	R wave axis (−4)	Heart rate (1068.8)	Sex (0.088)
9	Sex (−2)	Sex (147.1)	R wave axis (0.075)

**Fig. 4:**
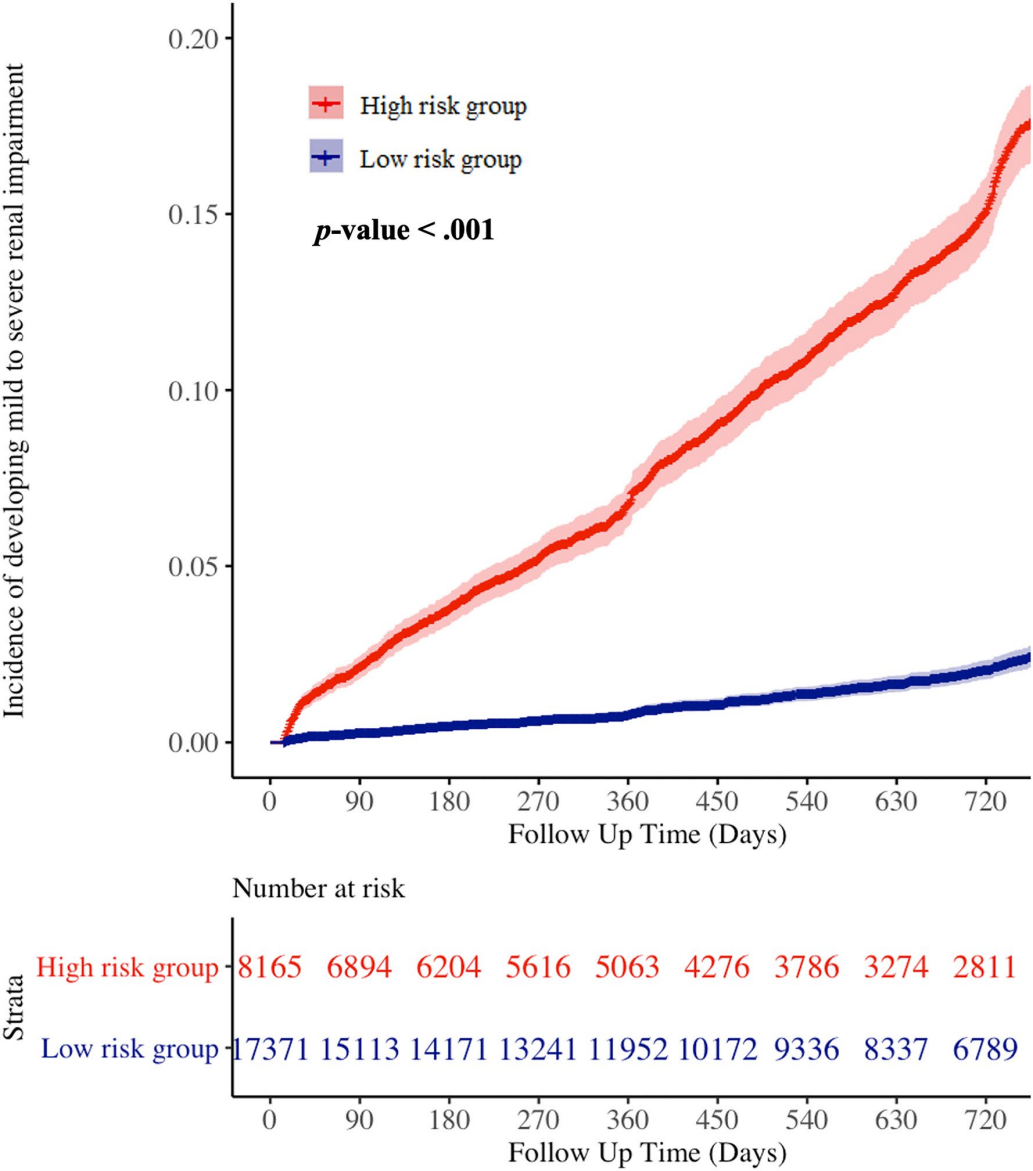
Cumulative risk of patients with no initial renal impairment developing renal impairment

Our study comprised 30,865 patients (22,949 and 7916 patients in the internal and external validation datasets, respectively) with follow-up laboratory results. Among them, 25,536 patients were normal (non-RI) at initial laboratory examination. We conducted a subgroup analysis of RI development after initial laboratory examination in these 25,536 patients, of whom 1,826 developed RI within 24 months. The high-risk group of the DLM demonstrated a significantly higher hazard (Fig. 4) and higher development rate of RI than the low-risk group (17.2% vs. 2.4%, respectively, *p* < 0.001).

## Discussion

We developed and validated a DLM based on an ensemble network for RI detection using 12-, six-, and single-lead ECGs and demonstrated reasonable performance. Subsequently, we visualized our DLM to determine the regions and characteristics of the ECG that were used for RI detection and verified the important variable for the decision in diverse statistical methods, such as logistic regression, random forest, and DLM. We conducted a subgroup analysis for patients with non-RI (normal) at the initial laboratory examination; it was demonstrated that the DLM could predict the development of RI. To our knowledge, this study is the first to develop a DLM for detecting and predicting RI and demonstrating interpretable patterns of decision making using the DLM. In a previous study, Rahman et al. showed the possibility that cardio-renal syndrome patients could be detected using ECG with a machine learning model (support vector machine). However, this study used data from a small population with renal disease. In our study, we developed a deep learning model (DLM) using big data and confirmed the accuracy using both internal and external validation datasets.

Developing a reliable screening tool for detecting and predicting RI is the cornerstone for early diagnosis of RI and preventing irreversible disease progression for end stage renal disease, which requires renal replacement treatment. Most RI patients were asymptomatic and had nonspecific symptoms. Diagnostic examinations are laboratory tests that require invasive blood sampling and cannot be conducted in daily living and low-income countries. Therefore, a new technology is required for detecting RI with simple and noninvasive methods that could be adopted in daily living. As ECG is a non-invasive test and is changed with RI, we developed a DLM for detecting RI using ECG.

The most important aspect of deep learning is its ability to extract features and develop an algorithm using various types of data, such as images, 2D data, and waveforms [33]. In previous studies, Attia and colleagues and our study group developed a DLM to screen for heart failure, arrhythmia, valvular heart disease, left ventricular hypertrophy, electrolyte imbalance, and anemia [16–24]. Deep learning is criticized for its unreliable outcomes because of the low transparency of the process, the so-called black box. Therefore, we adopted a sensitivity map to describe the abnormal findings that affected the decision of the DLM for detecting RI and describing the variable importance of ECG features. Using this method, we verified an ECG region and features that were associated with RI. In conventional methods, the research process began based on the hypothesis of researchers. For example, in the association between RI and ECG, researchers hypothesized based on their experience of dictating the ECG of RI. This method limited the opportunity to discover knowledge in human perception. In deep learning methods, such as DLM and sensitivity mapping in this study, the findings were not based on previous medical knowledge of humans, but on the data itself. Therefore, we had the opportunity to discover new knowledge from the data itself without human prejudice. Deep learning could discover the complex hierarchical non-linear representation that could not be discovered using conventional statistical methods, such as logistic regression. In this study, we verified the important ECG region for detecting RI from waveform data. We verified that RI could be detected and predicted using ECG based on a DLM. Further, we verified that specific ECG features, such as the QRS duration, T-wave axis, and corrected QT interval, were correlated with detecting RI. These findings were in agreement with the results of previous studies. Bignotto et al. and Stewart et al. demonstrated that left ventricular hypertrophy was identified in 50–80% of the RI population [34, 35]. Shafi et al. demonstrated that a widening of the QRS complex and prolonged QTc was identified in RI populations [36]. Deo et al. verified that ECG metrics, such as the PR interval, QRS duration, and QTc, were independent risk markers for cardiovascular death [13].

We developed and experimented DLMs using diverse format of ECG, such as 12, 6, and single lead ECG. We also showed the differences of AUC of the DLM for detecting RI using 12 lead ECG and other lead ECG during internal and external validation. Although the p-values for differences of AUC of the DLM for detecting a moderated to severe RI using 12 lead ECG and other leads ECG were <0.001, the effect sizes were 0.008–0.023. In medical big data research, since the *p*-value could be significant due to the large sample size, it is important to interpret results in consideration of the effect size.

There were several limitations to this study. First, we validated the DLM using retrospective data; however, it is necessary to validate DLM with prospective studies and data from daily living. Studies related to the clinical significance of the new technology are required for application in clinical practice. In our next study, we will verify DLM performance and significance with a prospective study in daily clinical practice. Second, this study was conducted only in two hospitals in Korea; hence, it is necessary to validate the DLM with patients in other countries. Third, although we compared the variable importance ranking in several machine learning and DLMs, we could not confirm the exact statistics between the importance results due to the lack of machine learning and deep learning statistical methods. Several improvements in the machine learning area helped find new methods for comparing the importance results more precisely.

## Conclusion

The DLM demonstrated accurate performance in detecting RI using ECG. The DLM successfully demonstrated the abnormality of ECG, which was correlated with RI. The application of artificial intelligence technologies based on the DLM to ECG could enable screening for RI and predict the development of RI.

## Supplementary Information

Below is the link to the electronic supplementary material.

Supplementary file1 (DOCX 108 KB)

## Data Availability

The data underlying this article will be shared on reasonable request to the corresponding author.

## Abbreviations

AUC: Area under the receiver operating characteristic curve
CI: Confidence interval
DLM: Deep learning model
ECG: Electrocardiography
eGFR: Estimated glomerular filtration rate
MSH: Mediplex Sejong Hospital
NPV: Negative predictive value
PPV: Positive predictive value
RI: Renal impairment
SD: Standard deviation
SGH: Sejong General Hospital
2D: 2 Dimensional
1D: 1 Dimensional

## Code availability

The data underlying this article will be shared on reasonable request to the corresponding author.

## References

[1] Himmelfarb J, Ikizler TA (2010). Hemodialysis. N Engl J Med.

[2] Liyanage T, Ninomiya T, Jha V, Neal B, Patrice HM, Okpechi I, Zhao M, Lv J, Garg AX, Knight J, Rodgers A, Gallagher M, Kotwal S, Cass A, Perkovic V (2015). Worldwide access to treatment for end-stage kidney disease: a systematic review. Lancet.

[3] Ruggenenti P, Cravedi P, Remuzzi G (2012). Mechanisms and treatment of CKD. J Am Soc Nephrol.

[4] Trivedi HS, Pang MMH, Campbell A, Saab P (2002). Slowing the progression of chronic renal failure: economic benefits and patients’ perspectives. Am J Kidney Dis.

[5] Webster AC, Nagler EV, Morton RL, Masson P (2017). Chronic kidney disease. Lancet.

[6] Chen TK, Knicely DH, Grams ME (2019). Chronic kidney disease diagnosis and management. JAMA.

[7] Stanifer JW, Jing B, Tolan S, Helmke N, Mukerjee R, Naicker S, Patel U (2014). The epidemiology of chronic kidney disease in sub-Saharan Africa: a systematic review and meta-analysis. Lancet Glob Heal.

[8] Dhondup T, Qian Q (2017). Acid-base and electrolyte disorders in patients with and without chronic kidney disease: an update. Kidney Dis.

[9] Hung S, Lai Y, Kuo K, Tarng D (2015). Volume overload and adverse outcomes in chronic kidney disease: clinical observational and animal studies. J Am Heart Assoc.

[10] McAlister FA, Ezekowitz J, Tonelli M, Armstrong PW (2004). Renal insufficiency and heart failure. Circulation.

[11] Sarnak MJ, Levey AS, Schoolwerth AC, Coresh J, Culleton B, Hamm LL, McCullough PA, Kasiske BL, Kelepouris E, Klag MJ, Parfrey P, Pfeffer M, Raij L, Spinosa DJ, Wilson PW (2003). Kidney disease as a risk factor for development of cardiovascular disease. Hypertension.

[12] Matsushita K, Coresh J, Sang Y, Chalmers J, Fox C, Guallar E, Jafar T, Jassal SK, Landman GWD, Muntner P, Roderick P, Sairenchi T, Schöttker B, Shankar A, Shlipak M, Tonelli M, Townend J, van Zuilen A, Yamagishi K, Yamashita K, Gansevoort R, Sarnak M, Warnock DG, Woodward M, Ärnlöv J (2015). Estimated glomerular filtration rate and albuminuria for prediction of cardiovascular outcomes: a collaborative meta-analysis of individual participant data. Lancet Diabetes Endocrinol.

[13] Deo R, Shou H, Soliman EZ, Yang W, Arkin JM, Zhang X, Townsend RR, Go AS, Shlipak MG, Feldman HI (2016). Electrocardiographic measures and prediction of cardiovascular and noncardiovascular death in CKD. J Am Soc Nephrol.

[14] Kestenbaum B, Rudser KD, Shlipak MG, Fried LF, Newman AB, Katz R, Sarnak MJ, Seliger S, Stehman-Breen C, Prineas R, Siscovick DS (2007). Kidney function, electrocardiographic findings, and cardiovascular events among older adults. Clin J Am Soc Nephrol.

[15] Dobre M, Brateanu A, Rashidi A, Rahman M (2012). Electrocardiogram abnormalities and cardiovascular mortality in elderly patients with CKD. Clin J Am Soc Nephrol.

[16] Attia ZI, Kapa S, Lopez-Jimenez F, McKie PM, Ladewig DJ, Satam G, Pellikka PA, Enriquez-Sarano M, Noseworthy PA, Munger TM, Asirvatham SJ, Scott CG, Carter RE, Friedman PA (2019). Screening for cardiac contractile dysfunction using an artificial intelligence–enabled electrocardiogram. Nat Med.

[17] Attia ZI, Friedman PA, Noseworthy PA, Lopez-Jimenez F, Ladewig DJ, Satam G, Pellikka PA, Munger TM, Asirvatham SJ, Scott CG, Carter RE, Kapa S (2019). Age and sex estimation using artificial intelligence from standard 12-lead ECGs. Circ. Arrhythmia Electrophysiol..

[18] Galloway CD, Valys AV, Shreibati JB, Treiman DL, Petterson FL, Gundotra VP, Albert DE, Attia ZI, Carter RE, Asirvatham SJ, Ackerman MJ, Noseworthy PA, Dillon JJ, Friedman PA (2019). Development and validation of a deep-learning model to screen for hyperkalemia from the electrocardiogram. JAMA Cardiol.

[19] Attia ZI, Noseworthy PA, Lopez-Jimenez F, Asirvatham SJ, Deshmukh AJ, Gersh BJ, Carter RE, Yao X, Rabinstein AA, Erickson BJ, Kapa S, Friedman PA (2019). An artificial intelligence-enabled ECG algorithm for the identification of patients with atrial fibrillation during sinus rhythm: a retrospective analysis of outcome prediction. Lancet.

[20] Cho Y, Kwon J-M, Kim K-H, Medina-Inojosa JR, Jeon K-H, Cho S, Lee SY, Park J, Oh B-H (2020). Artificial intelligence algorithm for detecting myocardial infarction using six-lead electrocardiography. Sci Rep.

[21] Jo Y-Y, Cho Y, Lee SY, Kwon J, Kim K-H, Jeon K-H, Cho S, Park J, Oh B-H (2020). Explainable artificial intelligence to detect atrial fibrillation using electrocardiogram. Int J Cardiol.

[22] Kwon J, Cho Y, Jeon K-H, Cho S, Kim K-H, Baek SD, Jeung S, Park J, Oh B-H (2020). A deep learning algorithm to detect anaemia with ECGs: a retrospective, multicentre study. Lancet Digit Heal.

[23] Kwon J, Lee SY, Jeon K, Lee Y, Kim K, Park J, Oh B, Lee M (2020). Deep learning-based algorithm for detecting aortic stenosis using electrocardiography. J Am Heart Assoc.

[24] Myoung Kwon J, Kim KH, Medina-Inojosa J, Jeon KH, Park J, Oh BH (2020). Artificial intelligence for early prediction of pulmonary hypertension using electrocardiography. J Hear Lung Transplant.

[25] Levey AS, Coresh J, Greene T, Stevens LA, Zhang Y, Hendriksen S, Kusek JW, Van Lente F (2006). Using Standardized serum creatinine values in the modification of diet in renal disease study equation for estimating glomerular filtration rate. Ann Intern Med.

[26] Cheung AK, Chang TI, Cushman WC, Furth SL, Hou FF, Ix JH, Knoll GA, Muntner P, Pecoits-Filho R, Sarnak MJ, Tobe SW, Tomson CRV, Mann JFE (2021). KDIGO 2021 clinical practice guideline for the management of blood pressure in chronic kidney disease. Kidney Int.

[27] Schisterman EF, Perkins NJ, Liu A, Bondell H (2005). Optimal cut-point and its corresponding Youden index to discriminate individuals using pooled blood samples. Epidemiology.

[28] Carpenter J, Bithell J (2000). Bootstrap confidence intervals: when, which, what? A practical guide for medical statisticians. Stat Med.

[29] Lai MHC (2021). Bootstrap confidence intervals for multilevel standardized effect size. Multivariate Behav Res.

[30] R.R. Selvaraju, M. Cogswell, A. Das, R. Vedantam, D. Parikh, D. Batra, Grad-CAM: visual explanations from deep networks via gradient-based localization, in: Proc. IEEE Int. Conf. Comput. Vis., 2017: pp. 1;618–626. 10.1109/ICCV.2017.74.

[31] Selvaraju RR, Cogswell M, Das A, Vedantam R, Parikh D, Batra D (2020). Grad-CAM: visual explanations from deep networks via gradient-based localization. Int J Comput Vis.

[32] Zhang Z, Beck MW, Winkler DA, Huang B, Sibanda W, Goyal H (2018). Opening the black box of neural networks: methods for interpreting neural network models in clinical applications. Ann Transl Med.

[33] LeCun Y, Bengio Y, Hinton G (2015). Deep learning. Nature.

[34] Bignotto LH, Kallás ME, Djouki RJT, Sassaki MM, Voss GO, Soto CL, Frattini F, Medeiros FSR (2012). Electrocardiographic findings in chronic hemodialysis patients. J Bras Nefrol.

[35] Stewart GA, Gansevoort RONT, Mark PB, Rooney E, Mcdonagh TA, Dargie HJ, Stuart R, Rodger C, Jardine AG (2005). Electrocardiographic abnormalities and uremic cardiomyopathy. Kidney Int.

[36] Shafi S, Saleem M, Anjum R, Abdullah W, Shafi T (2017). ECG abnormalities in patients with chronic kidney disease. J Ayub Med Coll Abbottabad.

