# Supplementary material for: Artificial intelligence assessment for early detection and prediction of renal impairment using electrocardiography
Source: Int Urol Nephrol. 2022 Apr 11;54(10):2733–44. doi: 10.1007/s11255-022-03165-w (PMC9463260; doi:10.1007/s11255-022-03165-w)
Supplement: Supplementary file 1 — Supplementary file1 (DOCX 108 KB) [file 11255_2022_3165_MOESM1_ESM.docx]

**Supplementary material**

**Supplementary Figure S1. Study flowchart**

****Legend: ECG denotes electrocardiography
